# Reverse Coding of a Common-Sense Physical Activity Intervention for Older Adults Using Elements of the Behaviour Change Wheel Framework

**DOI:** 10.1177/15248399221081832

**Published:** 2022-04-06

**Authors:** Andrew James Powell, Sarah Thomas

**Affiliations:** 1 Bournemouth University, Bournemouth, UK

**Keywords:** physical activity/exercise, behavior change, community intervention, program planning and evaluation, behavior change theory, theory

## Abstract

“Common-sense” physical activity (PA) interventions for older adults may be more effective if developed in accordance with behavior change theory. One way to achieve this is through retrospectively applying a theoretical behavior change framework to “reverse code” an existing intervention and guide its ongoing development. This study aimed to detail a clear and systematic procedure that applied elements of the Behaviour Change Wheel (BCW) framework to reverse code the Active Ageing Pathway (AAP) intervention. The objectives of the procedure were to characterize the content of the AAP and its links to behavior change theory. The content of the AAP was first deconstructed through the examination of “standard operating procedures” documents, in-person observation, and a series of face-to-face discussions with AAP management. Then, the behavior change techniques (BCT) and BCW intervention functions associated with the AAP’s content were identified and coded using the BCT Taxonomy version 1. Forty-one active components were identified within the AAP, which involved numerous professionals, and pertained to a diverse and interlinked range of factors, across various modes of delivery. The components were classified under 20 separate BCT labels, which related to eight of the nine BCW intervention functions. These outcomes were demonstrated to have practical applications for identifying gaps in intervention content as well as for guiding future intervention evaluation. This study supports previous work detailing the usefulness of reverse coding procedures as a tool for developing common-sense interventions, and is the first to do so in the context of a PA intervention for older adults.

## Background

Physical inactivity is a significant risk factor for the development of age-related ill health and long-term disease (Booth et al., 2011), and there is a wealth of evidence suggesting that participating in regular physical activity (PA) provides a multitude of preventive health and quality of life benefits for individuals as they reach middle-age and beyond (Paterson et al., 2007; Taylor et al., 2004). Although World Health Organization guidelines recommend that over 55s perform at least 150 minutes of moderate-intensity PA per week to obtain these benefits (World Health Organization, 2010), at present only 46% of such individuals in the United Kingdom are doing so (British Heart Foundation, 2015). Thus, increasing the PA levels of older adults has become a priority for public health interventions in the United Kingdom, to promote healthy aging and reduce the risk of preventable health conditions developing (Public Health England, 2014).

With uncertainty around the most effective intervention characteristics and components to increase older adults’ PA levels (Zubala et al., 2017), real-world public health practice often sees the implementation of “common-sense” PA interventions, which adopt “off-the-shelf” strategies. Although pragmatic and locally contextualized, there are concerns that these types of interventions have underdeveloped rationales for achieving outcomes, as they often do not consider the theory or evidence underpinning the behavior change strategies they adopt (Hansen et al., 2017; Michie et al., 2011). Furthermore, even when they are seemingly successful at an anecdotal level, they can be difficult to define, and their mechanisms of action and outcomes are subsequently hard to explain and measure (Watkins et al., 2016). This makes their evaluation, and potential implementation on a larger scale, and in novel settings, challenging.

These difficulties, along with the assertion that PA interventions for older adults are more likely to be effective in the long term if they are developed in accordance with behavior change theory (Hansen et al., 2017; Olanrewaju et al., 2016), have led to increasing support for a systematic, theory-driven approach to their design. One way this can be achieved is through the application of a behavior change framework, to support the detailed design, development, and characterization of an intervention, in terms of its content, theoretical rationale, and putative mechanisms of action (French et al., 2012; Steinmo et al., 2015).

Typically, the application of a behavior change framework occurs from the inception of the intervention design process. However, a more pragmatic approach with existing common-sense interventions is to use one retrospectively, to “reverse code” an intervention. Here, the objective is to systematically deconstruct the intervention to characterize its content and links to behavior change theory. This process can provide an understanding of the intervention’s theoretical underpinning and the strategies it uses to target behavior, which, in turn, can guide future evaluation and aid comparisons with other interventions. Furthermore, it can enable the identification of elements of the intervention that may need refining (Watkins et al., 2016).

**Figure 1:**
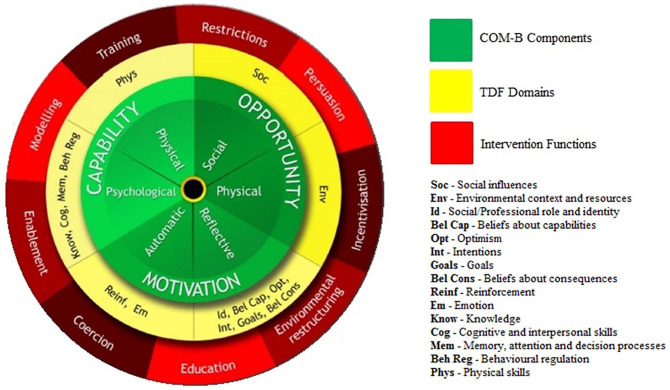
Behaviour Change Wheel (Michie et al., 2014; Smits et al., 2018). *Note.* This figure depicts the three layers of the Behaviour Change Wheel.

A well-established behavior change framework that can be used to reverse-code existing interventions is the Behaviour Change Wheel (BCW). The BCW integrates and synthesizes 19 other existing frameworks of behavior change into one unified model for developing interventions (Michie et al., 2014). The BCW comprises three layers (Figure 1). At its core is the Capability, Opportunity, Motivation, Behaviour (COM-B) model, which recognizes that changing behavior results from changing one or more components of psychological and/or physical capability, social and/or physical opportunity, and automatic and reflective motivation. The next layer is the Theoretical Domains Framework (TDF), which subdivides the components of the COM-B model and links them to 14 domains to provide a finer level of understanding. Surrounding the COM-B model and TDF is a layer of nine intervention functions. These are categories of mechanism, linked to the different COM-B components and TDF domains, by which an intervention can activate the theoretical pathways to influence behavior. Finally, there is a taxonomy of 93 behavior change techniques (BCT) associated with the BCW, which are the active components of an intervention that directly target behavior, and that link to the different intervention functions to ensure they are delivered (Michie et al., 2014; Smits et al., 2018).

As a relatively new approach, only a small number of studies have so far detailed and appraised different procedures for applying elements of the BCW framework to reverse code existing interventions, and none have done so in the context of a PA intervention for older adults. Steinmo et al. (2015) were the first to outline their process for identifying the BCTs and linked intervention functions of a “six steps of sepsis treatment” hospital implementation intervention, and mapping them to the TDF domains and corresponding COM-B conditions. They aimed to characterize the intervention’s content and potential theoretical mechanisms of action, and reported that their results provided a sound platform for intervention improvement and replication. Watkins et al. (2016) applied the COM-B model and identified the BCTs used in an asthma management intervention to examine the theoretical rationale behind its content, and were able to conclude that the intervention’s content had a sound theoretical underpinning. McHugh et al. (2018) identified the BCTs and linked intervention functions to characterize a multilevel implementation strategy for a fall prevention program, and reported that the process was useful for describing the intervention’s components and highlighting gaps that can be addressed to maximize effectiveness. Bourne et al. (2020) outlined a process that involved identifying the BCTs associated with an exercise intervention for individuals at risk of Type 2 diabetes and mapping them against the TDF. They concluded that their results increased the transparency of intervention content and enabled the targeted mechanisms of action to be identified, which could support future intervention improvements. Most recently, Pearson et al. (2020) identified the BCTs in a fibromyalgia self-management intervention and reported that their work provided an in-depth understanding of the intervention’s actions.

### Purpose

To add to the above evidence, the aim of this study is to detail a procedure that was used to apply elements of the BCW to reverse code an existing common-sense PA intervention for older adults. The objective of the procedure was to characterize the content of the intervention along with its links to behavior change theory, through deconstructing the intervention and identifying the associated BCTs and their related BCW intervention functions.

## Method

### The Intervention

The intervention reverse coded in this study was the Active Ageing Pathway (AAP), located in Dorset, South-West England. Originally implemented by local public health agency Active Dorset in March 2018, the AAP exists to provide individuals aged 55 and above with the knowledge, skills, and opportunities that will enable them to feel more confident in being regularly physically active and more able to embed this behavior into their everyday lives.

The AAP was initially developed by Active Dorset in response to Dorset’s 2015 “Sustainability and Transformation Plan” (Our Dorset, 2018), which highlighted anticipated population growth among the oldest people in the area with corresponding increases in long-term conditions, fragmentation, and variation in the quality of local health and care services, and the likelihood of future funding shortfalls. The aim of the AAP was therefore to bring together the different PA services in Dorset into one streamlined, standardized system, to deliver optimal behavioral and preventive health and wellbeing outcomes. Furthermore, the AAP sought to incorporate the relevant local National Health Service (NHS) and local authority organizations as “referrers” into this system, to deliver more efficient and effective partnership working and increased economic sustainability. The AAP is expected to grow and develop in the future to link up with community and workplace organizations and populations.

The AAP currently operates by engaging local secondary care clinicians and health care professionals to identify inactive individuals aged 55 and above during routine clinical appointments as part of their standard patient treatment pathways. Individuals are then invited to attend group “Wellbeing Events” run by Active Dorset. These last for 3 hours, and aim to educate attendees on the benefits of becoming more active, and showcase the PA options available locally, ranging from gym-based exercise referral schemes and organized walking clubs to tai-chi classes. Following attendance at a Wellbeing Event, individuals are then invited by Active Dorset to sign up to the LiveWell Dorset (LWD) integrated lifestyle service. Here, they can receive 6 weeks of behavior change coaching support online and via telephone from a specially trained “Wellness Coach,” to help them to meet the Government’s recommended guidelines for PA, and then maintain this lifestyle change.

As part of a Sport England-funded project, Active Dorset wished to find out about the elements of the AAP that influence and support individuals to change their PA behavior, in terms of specific BCTs, interpersonal approaches, and service pathway design. The reverse coding of the AAP was the first part of that work, and it was hoped that the outcomes would underpin further project work to evaluate the AAP, as well as guide the future optimization of its content.

### Procedure

Drawing upon previous work (Bourne et al., 2020; McHugh et al., 2018; Pearson et al., 2020; Steinmo et al., 2015; Watkins et al., 2016), a two-step procedure was followed to reverse code the AAP.

#### Step 1—Deconstructing Intervention Content

In order to characterize the AAP’s content, its active components, along with an understanding of their rationale for inclusion in the AAP, were deconstructed by the lead author (AJP) through the examination of “standard operating procedures” documents provided by Active Dorset, in-person observation of Wellbeing Events, and a series of face-to-face discussions with Active Dorset management. Components of the AAP were identified on the basis of being observable, replicable, and irreducible, and explicitly linking to both the target behavior and the target population of the AAP (Michie et al., 2014). The standard operating procedures documents supplied by Active Dorset contained detailed intervention scripts and content descriptions. AJP read and re-read the documents and took notes on them. Observations of Wellbeing Events were carried out on three separate occasions. AJP made detailed field notes on the content of the events while in attendance. Face-to-face discussions with Active Dorset management mainly served to clarify the information that had been gained from the standard operating procedures documents and observation of Wellbeing Events. AJP also took notes during these meetings.

#### Step 2—Linking Intervention Content to Behavior Change Theory

Once all intervention components had been identified, their associated BCTs and related intervention functions were then coded by AJP, to characterize the links of the AAP’s content to behavior change theory. Using the BCT Taxonomy version 1 (Michie et al., 2014), each AAP component was checked against BCT definitions and labeled with the BCT/s deemed most representative of its perceived purpose within the AAP. Once all BCT labels had been assigned, each AAP component was then categorized with the BCW intervention function/s corresponding to its BCT label/s (Michie et al., 2014). All BCT labels were checked by the co-author (ST), with disagreements on any labels resolved by discussion and consensus. This measure was taken due to the recognition that labeling of BCTs can be subjectively influenced by both the richness of content description and varying broadness of BCT definitions (Smits et al., 2018). Both authors had undertaken online training in BCT coding prior to starting the labeling process.

## Results

A summary of the deconstructed AAP with its composite components and their rationale, BCT labels, and corresponding intervention functions is shown in Table 1.

**Table 1:** Summary of AAP Components and Their Rationale, BCT Labels, and Corresponding Intervention Functions (Michie et al., 2014)

Intervention component	Rationale for component	BCT/s	Intervention function/s
Clinician/health professional conveys information about AAP to individual	To facilitate awareness of AAP	Social support (unspecified)	Enablement
Clinician/health professional conveys information about Wellbeing Event to individual	To facilitate awareness of Wellbeing Event	Social support (unspecified)	Enablement
Clinician/health professional conveys information on benefits of physical activity to individual	To facilitate knowledge on benefits of physical activity	Information about health consequences	Education Persuasion
Clinician/health professional provides leaflet about Wellbeing Event to individual	To facilitate awareness of Wellbeing Event	Social support (unspecified)	Enablement
Clinician/health professional conveys information about Wellbeing Event sign-up process to individual	To facilitate registration for Wellbeing Event	Social support (unspecified)	Enablement
Simple web-based sign-up process provided for individual to register to attend next Wellbeing Event	To facilitate registration for Wellbeing Event	Adding objects to the environment	Enablement Environmental restructuring
Clinician/health professional provides help to individual to complete online Wellbeing Event registration, or completes it for them, if required	To facilitate registration for Wellbeing Event	Social support (practical)	Enablement
Clinician/health professional sends reminder leaflet to individual 1 month prior to next Wellbeing Event	To facilitate registration for Wellbeing Event	Prompts/cues Social support (practical)	Education Enablement Environmental restructuring
Registration website provides information and details on Wellbeing Event for individual	To facilitate awareness of Wellbeing Event	Social support (unspecified)	Enablement
Registration website offers option for individual to bring guests to Wellbeing Event	To facilitate registration and attendance of Wellbeing Event	Adding objects to the environment Social support (emotional)	Enablement Environmental restructuring
Wellbeing Event held at venue with ‘favorable’ characteristics: • Venue is local landmark/well-known • Venue is nonmedical • Venue is modern and pleasant • Venue has plentiful parking • Venue has good public transport links • Venue is in easy-to-access central location	To facilitate attendance of Wellbeing Event	Restructuring the physical environment	Enablement Environmental restructuring
Wellbeing Event scheduled during off-peak daylight hours	To facilitate attendance of Wellbeing Event	Restructuring the physical environment	Enablement Environmental restructuring
Wellbeing Event free to attend	To facilitate attendance of Wellbeing Event	Restructuring the physical environment	Enablement Environmental restructuring
Wellbeing Event contains scheduled time for “coffee and mingling” where group is encouraged to interact and share experiences with one another	To facilitate awareness of others’ behavior to allow comparison with own	Social comparison Social support (unspecified)	Enablement Persuasion
Wellbeing Event contains exhibition area with stalls advertising local physical activity services and providing opportunities to register	To facilitate awareness of local physical activity opportunities	Adding objects to the environment Social support (practical) Social support (unspecified)	Enablement Environmental restructuring
Fitness instructor presents information about general well-being and health benefits of physical activity to group	To facilitate knowledge on wellbeing	Credible source Information about health consequences	Education Persuasion
Fitness instructor instructs group to perform 3-minute “movement” session to convey how physical activity positively affects feelings of wellbeing	To facilitate awareness of feelings generated by physical activity	Demonstration of the behavior Monitoring of emotional consequences	Enablement Modeling Training
Fitness instructor presents technical information about physical activity (e.g., frequency, duration, intensity, mode) and safety/risk considerations to group	To facilitate knowledge on how to effectively perform physical activity	Instruction on how to perform a behavior Social support (unspecified)	Enablement Training
Fitness instructor presents simple examples of how people can get started with physical activity at home on their own to group	To facilitate knowledge on practical ways to perform physical activity	Instruction on how to perform a behavior	Training
Fitness instructor interactively discusses common barriers and facilitators of physical activity with group	To facilitate analysis of factors influencing behavior and strategies to overcome them	Problem-solving	Enablement
Fitness instructor provides written materials and leaflets about physical activity to group	To facilitate knowledge on physical activity	Social support (unspecified)	Enablement
Representatives from stalls advertising local physical activity services make “pitches” to group about their offerings, including details on how to register	To facilitate registration for local physical activity opportunities	Social support (unspecified)	Enablement
Wellness Coach presents information about LiveWell Dorset service to group	To facilitate awareness of LiveWell Dorset service	Social support (unspecified)	Enablement
Wellness Coach presents information about benefits of behavior change coaching to group	To facilitate knowledge on benefits of coaching	Information about health consequences	Education Persuasion
Wellness Coach presents information about LiveWell Dorset service registration processes to group	To facilitate registration for LiveWell Dorset service	Social support (unspecified)	Enablement
Wellness Coach provides cards with information on how to register with LiveWell Dorset service to group	To facilitate registration for LiveWell Dorset service	Social support (unspecified)	Enablement
Wellness Coach provides opportunity for group to provide personal details in order to receive a “registration call-back” from LiveWell Dorset service	To facilitate registration for LiveWell Dorset service	Adding objects to the environment Social support (practical)	Enablement Environmental restructuring
Simple telephone and web-based chat facilities provided for individual to enquire about Live Well Dorset service	To facilitate registration for LiveWell Dorset service	Adding objects to the environment Social support (unspecified)	Enablement Environmental restructuring
Simple telephone and web-based sign-up processes provided for individual to register with LiveWell Dorset service	To facilitate registration for LiveWell Dorset service	Adding objects to the environment	Enablement Environmental restructuring
Wellness Advisor conducts introductory LiveWell Dorset assessment with individual via telephone (and follow-up assessments at 3, 6, and 12 months after introductory assessment), completing and discussing current lifestyle and physical activity habits questionnaires with individual	To monitor and provide evaluative feedback on behavior	Feedback on behavior	Education Coercion Incentivization Persuasion Training
Wellness Advisor discusses benefits of physical activity with individual	To facilitate knowledge on benefits of physical activity	Information about health consequences	Education Persuasion
Wellness Advisor introduces web-based “local activity finder” tool to individual to help identify suitable physical activity opportunities to access	To facilitate awareness of local physical activity opportunities	Adding objects to the environment	Enablement Environmental restructuring
Wellness Advisor explores likely pros and cons with individual of accessing behavior change coaching	To facilitate consideration of possible positive benefits and outcomes of coaching	Comparative imagining of future outcomes Pros and cons	Enablement
Wellness Coach conducts weekly 20-minute coaching session with individual via telephone for 6 weeks	To provide general support for behavior	Social support (unspecified)	Enablement
Wellness Coach sets physical activity goals with individual over course of 6 weeks	To facilitate setting of goals in terms of behaviors to be achieved	Goal setting (behavior)	Enablement
Wellness Coach reviews physical activity goals with individual over course of 6 weeks	To facilitate reviewing of goals and consideration of modifications if necessary	Review behavior goal(s)	Enablement
Wellness Coach sets and reviews physical activity action plans with individual over course of 6 weeks	To facilitate detailed planning of behavior	Action planning	Enablement
Wellness Coach explores problems and challenges to becoming more physically active with individual over course of 6 weeks	To facilitate analysis of factors influencing behavior and strategies to overcome them	Problem-solving	Enablement
Wellness Coach discusses progress with individual over course of 6 weeks	To monitor and provide informative and evaluative feedback on behavior	Feedback on behavior	Coercion Education Incentivization Persuasion Training
Wellness Coach uses web-based ‘physical activity tracker’ tool with individual to record activities in between coaching sessions over course of 6 weeks	To facilitate monitoring and recording of behavior	Adding objects to the environment Self-monitoring of behavior	Coercion Education Enablement Environmental restructuring Incentivization Training
Wellness Coach explores likely pros and cons with individual at end of 6-week behavior change coaching course of accessing another 6-week round of coaching	To facilitate consideration of possible positive benefits and outcomes of coaching	Comparative imagining of future outcomes Pros and cons	Enablement

*Note*. AAP = Active Ageing Pathway; BCT = behavior change technique.

### Step 1—Deconstructing Intervention Content

Forty-one active components were identified within the AAP. The 41 components pertained to a diverse and interlinked range of factors, across face-to-face, group, telephone, and online contexts, and involved numerous professionals, including clinicians and health care professionals, fitness instructors, and behavior change coaches. In summary, the content of the AAP was tailored toward making individuals aware of the AAP and then facilitating them to attend a Wellbeing Event to learn about the benefits of PA and local opportunities available to them, and to then join the LWD service to receive tailored behavioral support to increase their PA levels while accessing these opportunities. Identified AAP components appeared to focus on either supporting participants’ uptake and progression through the AAP (e.g., providing a simple web-based Wellbeing Event registration process, holding Wellbeing Events at “favorable” venues and times of day) or directly influencing individuals’ PA behavior (e.g., exploring the problems and challenges to becoming more active, conveying information about the health benefits of PA).

### Step 2—Linking Intervention Content to Behavior Change Theory

The 41 components of the AAP were classified under 20 separate BCT labels, which in turn corresponded to eight of the nine BCW intervention functions. The most common BCTs were *social support (unspecified)*, *adding objects to the environment, information about health consequences, social support (practical)*, and *restructuring the physical environment*. The most common intervention functions were *enablement*, *environmental restructuring*, *education, persuasion*, and *training*. The intervention function that did not relate to any AAP components was *restriction*, while only one AAP component related to the *modeling* function. Therefore, to summarize, the AAP largely attempted to modify the physical or social context, use communication to stimulate action, and increase participants’ skills, knowledge, understanding, capability, and opportunity, to achieve its intended objectives (Michie et al., 2014). Table 2 provides an overall summary of the BCTs and corresponding intervention functions linked to the content of the AAP.

**Table 2:** Summary of BCTs and Corresponding Intervention Functions Linked to the Content of the AAP (Michie et al., 2014)

BCT	Corresponding intervention function/s
Social support (unspecified) Adding objects to the environment Information about health consequences Social support (practical) Restructuring the physical environment Comparative imagining of future outcomes Instruction on how to perform a behavior Feedback on behavior Problem-solving Pros and cons Action planning Credible source Demonstration of the behavior Goal setting (behavior) Monitoring of emotional consequences Prompts/cues Review behavior goal(s) Self-monitoring of behavior Social comparison Social support (emotional)	Enablement Enablement; environmental restructuring Education; persuasion Enablement Enablement; environmental restructuring Enablement Training Coercion; education; incentivization; persuasion; training Enablement Enablement Enablement Persuasion Modeling Enablement Enablement Education; environmental restructuring Enablement Coercion; education; enablement; incentivization; training Persuasion Enablement

*Note*. AAP = Active Ageing Pathway; BCT = behavior change techniques.

## Discussion

This study has detailed a procedure that was used to apply elements of the BCW framework to reverse code the AAP, an existing common-sense PA intervention for older adults. The content of the AAP was deconstructed, before the associated BCTs and related intervention functions were identified.

The procedure achieved its first objective, which was to characterize the content of the AAP. Through its deconstruction, 41 active components were identified within the AAP, which involved numerous professionals,and pertained to a diverse and interlinked range of factors, across face-to-face, group, telephone, and online modes of delivery. The AAP consisted of three elements, namely, making individuals aware of the pathway, facilitating their attendance at a Wellbeing Event, and subsequently joining the LWD service to receive direct behavioral support to increase their PA levels. Characterizing the AAP served to provide its operators Active Dorset with an in-depth breakdown of their intervention, arguably the first step toward them determining the specific elements that influence and support individuals to increase their PA levels through their Sport England-funded project. As initially intended, this information has subsequently been used by the authors to guide a qualitative research study focused specifically on participants’ experiences of the LWD service. Relating this to previous work, McHugh et al. (2018) similarly reported that the reverse coding procedure they used to characterize a falls prevention intervention provided them with a detailed understanding of the intervention’s content, which subsequently underpinned further research to explore its content and function in more depth and identify barriers to its success. Furthermore, Steinmo et al. (2015) stated that their procedure for characterizing a “six steps of sepsis treatment” hospital implementation intervention allowed them to systematically describe the intervention in a common language, and that they planned to use their understanding of its content to guide the subsequent evaluation of health professionals’ experiences of the intervention.

The current procedure also achieved its second objective, to characterize the links of the AAPs content to behavior change theory. The 41 active components of the AAP were classified under 20 separate BCT labels, which related to eight of the nine BCW intervention functions. The content of the AAP largely served to modify the physical or social context, use communication to stimulate action, and increase participants’ skills, knowledge, understanding, capability, and opportunity, to influence PA behavior. Previous reviews on interventions to increase the PA levels of older adults have not found consistent evidence for the effectiveness of particular BCTs (Sansano-Nadal et al., 2019; Zubala et al., 2017). However, it has been concluded that effective PA interventions typically incorporate greater numbers of BCTs and utilize a blend of behavioral, motivational, and/or cognitive methods to influence PA behavior (McEwan et al., 2019; Zubala et al., 2017). Characterizing the AAP’s links to behavior change theory confirmed to Active Dorset that the AAP appears to do this. Characterizing the AAP’s links to behavior change theory also had practical value for Active Dorset, namely through highlighting that the *restriction* and *modeling* intervention functions were underserved by its content. That the *restriction* intervention function was not linked to the AAP was seen as understandable; it tends to link to strategies that use the external environment to limit people’s behavior, whereas the focus of the AAP is on changing the way that people think, feel and react (Michie et al., 2014). However, the fact that only one AAP component linked to the *modeling* function was viewed as surprising, given that the purpose of this function (to provide a behavioral example for people to aspire to or imitate) is arguably crucial to the objectives of the AAP (Michie et al., 2014). This information, therefore, provided an indication that more content could potentially be added to the AAP serving the *modeling* function, focused on providing behavioral examples for participants to follow. Strategies subsequently considered for this purpose included adding extended fitness instructor-led PA sessions to Wellbeing Events and providing a web-based digital PA program for people to access at home. The practical usefulness of the current procedure again supports the findings of previous work. For instance, McHugh et al. (2018) reported that their aforementioned procedure helped to highlight gaps in intervention content that could be refined to maximize its effectiveness. Similarly, Watkins et al. (2016), who examined the theoretical rationale behind the content of an asthma management intervention, found that their reverse coding procedure provided a foundation to support intervention improvement.

### Limitations

Some limitations of the current study should be noted at this point. First, the content of the AAP was deconstructed through the examination of standard operating procedures documents, in-person observation of Wellbeing Events, and a series of face-to-face discussions with Active Dorset management. A more structured approach, such as conducting focus group interviews with both AAP participants and AAP professionals, may have provided more detailed and nuanced insights into the AAP’s content. Furthermore, only the primary author was involved in deconstructing the AAP’s content. Given the vast and dynamic nature of the AAP, and the number of actors and settings involved (McHugh et al., 2018), it is feasible that some components may have been missed. The involvement of at least one additional research team member in the deconstruction process might have helped to ensure that the most complete and accurate picture of the AAP’s content emerged. However, these limitations were largely a reflection of resource constraints, and a pragmatic approach was taken. Funding and capacity are often factors that limit the application and transferability of behavior change theory to “real-world” interventions (Hansen et al., 2017). This study, therefore, highlights the importance of adapting and tailoring reverse coding procedures to the available resources, while still retaining a structured, systematic approach.

### Implications for Research and Practice

PA interventions for older adults have become a priority public health focus in the United Kingdom as a means of promoting healthy aging and reducing the risk of preventable health conditions developing (Public Health England, 2014). However, with uncertainty around the most effective intervention characteristics and components to increase older adults’ PA levels (Zubala et al., 2017), common-sense PA interventions are often implemented, which adopt off-the-shelf strategies. It has been asserted that these interventions are more likely to be effective in the long term if they are developed in accordance with behavior change theory (Hansen et al., 2017; Olanrewaju et al., 2016), and one way this can be achieved is through the retrospective application of a theoretical behavior change framework to “reverse code” an intervention and guide its ongoing development (French et al., 2012; Steinmo et al., 2015). The main implications of the current study are, therefore, that

- a clear, systematic, and replicable procedure for applying elements of the BCW framework to reverse code an existing common-sense PA intervention for older adults was demonstrated.
- the procedure provided a detailed characterization of the intervention’s content and the links to behavior change theory, adding to the findings of previous research in the area.
- the procedure also demonstrated a practical application for identifying gaps in intervention content and guiding future intervention evaluation.

Common-sense PA interventions for older adults are thought to have underdeveloped rationales for achieving effectiveness, through not considering the theory or evidence underpinning the behavior change strategies they adopt (Hansen et al., 2017; Michie et al., 2011). It is often said to be difficult to define their content and mechanisms of action, and to measure and explain their outcomes, making them challenging to both evaluate and replicate in novel settings (Watkins et al., 2016). Therefore, procedures like the one demonstrated in this study arguably offer an important tool to overcome some of these problems, and a first step toward developing common-sense PA interventions into theory-linked ones that achieve the best possible outcomes.
